# Identification of Different Donor-Acceptor Structures via Förster Resonance Energy Transfer (FRET) in Quantum-Dot-Perylene Bisimide Assemblies

**DOI:** 10.3390/ijms10125239

**Published:** 2009-12-01

**Authors:** Danny Kowerko, Stefan Krause, Nicole Amecke, Mohamed Abdel-Mottaleb, Jörg Schuster, Christian von Borczyskowski

**Affiliations:** 1 Institute of Physics and nanoMA (Center for nanostructured Materials and Analytics), University of Technology, 09107 Chemnitz, Germany; E-Mail: borczyskowski@physik.tu-chemnitz.de (C.B.); 2 Institute of Experimental Physics I, Leipzig University, D-04103 Leipzig, Germany; 3 Sabry Corporation, Cairo, Egypt

**Keywords:** semiconductor nanocrystals, self assembly, fluorescence resonance energy transfer, perylene bisimide, single molecule

## Abstract

Nanoassemblies are formed via self-assembly of ZnS capped CdSe quantum dots (QD) and perylene bisimide (PBI) dyes. Upon assembly formation the QD photoluminescence is quenched, as can be detected both via single particle detection and ensemble experiments in solution. Quenching has been assigned to FRET and NON-FRET processes. Analysis of FRET allows for a distinction between different geometries of the QD dye assemblies. Time-resolved single molecule spectroscopy reveals intrinsic fluctuations of the PBI fluorescence lifetime and spectrum, caused by rearrangement of the phenoxy side groups. The distribution of such molecular conformations and their changed dynamics upon assembly formation are discussed in the scope of FRET efficiency and surface ligand density.

## Preface

In recent years we (http://www.tu-chemnitz.de/physik/OSMP/) have studied photophysics of (single) organic dye molecules and semiconductor nanocrystals [quantum dots (QD)] with special regard to (spectral) diffusion [1] and luminescence intermittency (blinking) of single molecules or particles [2]. Furthermore QD are used as donors in self-assembled aggregates with organic dye molecules as acceptor for Förster Resonance Energy Transfer (FRET) [3]. In such assemblies a new dye induced luminescence quenching mechanism has been identified and related to the tunnelling of the quantum-confined exciton wave function to the QD surface [4]. We recently quantified this non-FRET type quenching on a single particle level with functionalized perylene bisimide molecules [5].

In this paper we report how FRET can be used as a tool to provide insight into aggregate geometry of QD-dye assemblies, but also discuss typical FRET efficiencies affecting mechanisms like spectral shifts of the dye and the QD caused by conformational dynamics [6] or photooxidation, respectively.

## 1. Introduction

Much of the current research related to colloidal semiconductor quantum dots (QD) has been focused on photoinduced excitation energy transfer [7] processes. Of considerable importance are transfer processes between semiconductor materials and molecular dye species, since they offer versatile applications either as optical markers in complex biological systems [8–11] or for optimization of photovoltaic devices [12,13].

In many cases formation of QD-dye nanoassemblies is followed by QD photoluminescence quenching due to Förster-type resonant excitation energy transfer (FRET) [14–18], which is accompanied by enhancement of the dye fluorescence. Basically three strategies have been implemented in order to realize QD-dye assemblies:

1. Blends of QD-dye moieties have been used to realize photoactive devices [19,20]. Since this is a kind of macroscopic approach, not much is known about the microscopic realization of the involved assemblies.
2. The attachment of dye molecules has been accomplished via chemical bonds to a polymer shell covering the QD surface [21]. This results in timely and spatially fixed donor acceptor complexes, which are well defined [7] but with a relatively low FRET efficiency [22] since donor-acceptor distances are relatively large. Thus more than one dye per QD is needed to achieve high FRET efficiencies. Since QD-dye assemblies are possible candidates to monitor e.g., biological processes [24], such systems might not be optimal sensors to detect processes on the base of a single event.
3. The third approach is related to self-organized QD-dye assemblies via suitable functional groups of the dyes, which can anchor via “ligand-type” bonds to surface atoms of the QD. This approach is related to a dynamic process [3,4,25], which takes place as a competition between ligand bonding (e.g., of TOPO) and dye bonding This implies that QD-dye assemblies are not permanent in time, but might be nevertheless effective FRET systems on a single dye/single QD level. Such a dynamic approach, however, is often accompanied by NON-FRET photoluminescence (PL) quenching [5], which might be even larger than the influence of FRET itself [3]. This process is related to the excitonic wave function outside of the QD. The wavefunction will be disturbed by the dye attachment finally resulting in (self-) localization of the electron close to the surface or in modification of surface states giving rise to non-radiative decay [4]. On the other hand, this Non-FRET quenching mechanism might also be used as a monitor for dye related properties, like their functional group specific complexation constants. As an example, ortho-pyridyl free base porphyrins did not result in QD PL quenching while para- and meta-pyridyl freebase porphyrins have been shown to be strong quenchers. However, in case of properly selected ligands (class (iii)), rather stable QD-dye assemblies are formed [3].

The outcome of previous analysis is that for exact discrimination of FRET and Non-FRET mechanism, such assemblies need simultaneous quantitative investigation of QD PL quenching and dye fluorescence enhancement. This is often missing in published reports repeatedly resulting in incorrect assignments of processes and erroneous data evaluation.

The principle of molecular self assembly was transcribed to nano-assemblies of CdSe QD and pyridyl functionalized perylene bisimide (PBI) dye molecules, offering the investigation by single molecule spectroscopy. The observation of QD PL quenching caused by FRET and Non-FRET processes was analyzed, even on a single assembly level. The quantification and discrimination against further quenching mechanism has been extensively elucidated in [5].

In this paper we will not focus on PL quenching processes in ZnS capped CdSe quantum dots induced by functionalized PBI dyes. Instead, we exploit FRET related dye enhancement to access information about distinct nano-assembly geometries. Different attachment angles of PBI molecules coordinating to the ZnS shell of the QD are accomplished by pyridyl and terpyridyl substituted PBI molecules. Additionally, it undergoes intrinsic conformation dynamics [6,27], which are - depending on the environment - accompanied by spectral shifts up to 60 nm. Both the angle and the conformation of PBI realized on the CdSe/ZnS surface will in principle influence FRET efficiencies. Conformation dynamics, on the other hand, are used as probe for the surface coverage by ligands close to the dye.

With these investigations we aim at a microscopic understanding of the geometry and dynamics of QD-dye nanoassemblies. Moreover, luminescence intermittency (blinking, commonly considered to be related to photoinduced charge transfer) and spectral diffusion are identified on a single particle/single molecule level. Hence, we demonstrate how Förster theory based calculations of FRET efficiencies are used (i) to obtain information about surface geometry and (ii) to explain reduced FRET efficiencies at a single emitter level.

## 2. Results and Discussion

### 2.1. Ensemble Experiments

**Figure 1.**
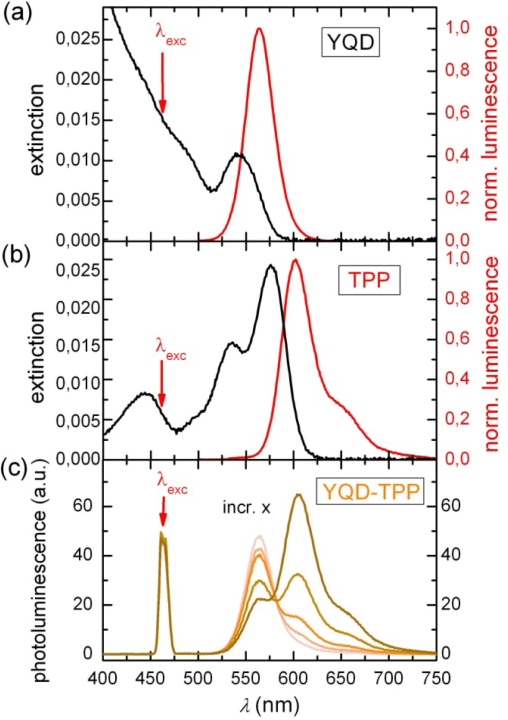
Absorption and emission spectra of (a) 0.1 μM YQD and (b) 0.45 μM TPP dissolved in toluene (λ_exc_ = 465 nm) at room temperature. **(c)** Emission spectra of YQD-TPP upon titration by TPP, which results in a decrease of YQD and an increase of TPP.

Figures 1a and 1b show the absorption and emission spectra of “yellow” (λ_em_ = 564 nm) CdSe/ZnS quantum dots (YQD) and terpyridyl perylene bisimide (TPP) as a prototype PBI dye (see Figure 10 for details). Optical spectra of the different PBI dyes are almost identical, whereas Figure 1c depicts the luminescence spectra of YQD-PP in toluene solution and the respective changes in intensity as a function of the relative molar ratio x of PBI to YQD. We have used two types of surfactants for YQD, namely a long chain amine (AM) and trioctylphosphine oxide (TOPO). Therefore we will discuss the respective YQD photoluminescence properties separately, since they differ considerably from each other. While TOPO surfactants result in nearly stable QD PL intensities (remaining constant during several hours after sample preparation as has also been observed in previous experiments [3]), the AM terminated QD PL intensities vary in time.

Titration of a 10^−7^ M YQD solution by PBI dyes results in an immediate PL quenching of YQD. Pyridyl substituents are known to be responsible for coordinating dye molecules to a QD surface [3]. In recently reported experiments three mechanisms for PL quenching following QD-dye assembly formation have been identified, namely Förster-type fluorescence resonance energy transfer (FRET) from a QD to dye molecules [7,21–24], photo-induced charge transfer (CT) from a QD to dyes [28–30] or vice versa [7] and a less specific mechanism which has been assigned to dye induced charge trapping or formation of surface states [4,31,32]. To identify the mechanisms responsible for quenching in the present experiments we have besides the PL of the QD also investigated the fluorescence of the dyes as a function of the molar ratio x. In case of FRET from a QD donor to a dye acceptor the dye fluorescence intensity should increase in case of assembly formation as compared to a free dye molecule at the same concentration. The FRET efficiency E_FRET_ related to the QD donor (D) and the dye acceptor (A) can be determined according to [33]:

(1) $$E_{FRET}=\frac{\varepsilon_{A}(F_{AD}-F_{A})}{\varepsilon_{D}F_{A}}\frac{1}{f_{AD}}$$

where ɛ corresponds to the respective extinction coefficients at the excitation wavelength (of 465 nm), F_AD_ to the acceptor fluorescence intensity in the presence and F_A_ in the absence of the donor D, while the fractional labeling f_AD_ gives the ratio of assembled to the total number of acceptor molecules. Equation (1) can be rearranged with regard to the acceptor enhancement Φ^EA^ by:

(2) $$\Phi^{EA}=\frac{\varepsilon_{A}(F_{AD}-F_{A})}{\varepsilon_{D}F_{A}}=E_{FRET}\;f_{AD}(x)$$

**Figure 2.**
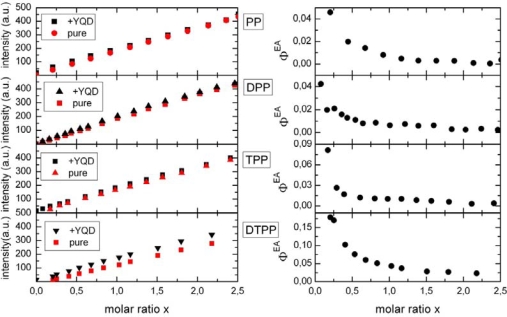
Fluorescence enhancement of PBI dyes by YQD. The left side shows the dye fluorescence intensities F without (pure) and as a function of the molar ratio x (c_PBI_/c_YQD_), whereat c_YQD_ was kept constant at 100 nM. On the right side we have plotted Φ^EA^ (Equation 2).

The efficiency of Förster type energy transfer from a donor coordinated to exactly one acceptor can be calculated according to Equation (3) and is a priori not a function of the molar ratio x as described later in the text. Since f_AD_ is a function of the acceptor concentration and hence a function of molar ratio, the observed Φ^EA^ reveals the fractional labeling f_AD_ as long as formation of donors with more than one acceptor can be neglected. In Figure 2 fluorescence intensities at 605 nm of four different PBI dyes with and without the presence of YQD (with TOPO ligands) is shown (left side).

It can be clearly seen that the YQD donor enhances the PBI fluorescence. The enhancement varies with the type of PBI. Similar results are observed in case of AM surfactants. However, the absolute enhancement is somewhat smaller as compared to TOPO surfactants [5]. We have plotted Φ^EA^ as a function of x. The enhancement is at most 17% for DTPP and close to 4% for PP.

**Figure 3.**
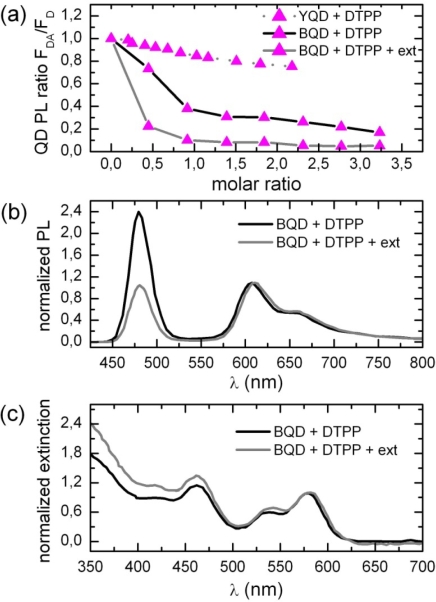
(a) PL quenching of (YQD-TOPO) and (BQD-TOPO) by titration with DTPP. The lowest curve has been obtained after extraction of excess TOPO from the toluene solution. Guide lines are shown only for the eye. (b) BQD-DTPP fluorescence and (c) absorption are shown for BQD before and after TOPO extraction. DTPP spectra have been normalized to the respective maxima.

To prove that assembly formation is accomplished by the functional groups a spectral red shift of terpyridyl substituted PBI is expected upon the coordination to metal ions, according to [42]. For a better deconvolution of the PBI and QD spectra, titration experiments were carried out with “blue” quantum dots (BQD). In Figure 3 (a) we have plotted the PL intensity as a function of the molar ratio x.

As expected, quenching is much more efficient as compared to YQD [4]. The formation of assemblies is hindered by the ligand shell for steric reasons, especially for comparably large dye molecules like DTPP (see Figure 10). This implies that most TPP are “free” and thus no real shift is observable. However, extracting TOPO from the toluene solution creates more vacant surface sites for DTPP attachment and thus enhances PL quenching considerably [3]. For BQD we obtain at x = 1 a quenching efficiency of nearly 100%. Under these conditions a small but reproducible red-shift of the DTPP fluorescence and absorption spectra of a few nm (see Figure 3) is present. The PL quenching by DTPP is for x = 0.5 even larger than one would expect for a 1:1 complex. This might be caused either by ligand loss as observed for other ligands [25] or by the fact, that one DTPP molecule might complex two BQD.

### 2.2. Single Particle/Single Molecule Experiments

**Figure 4.**
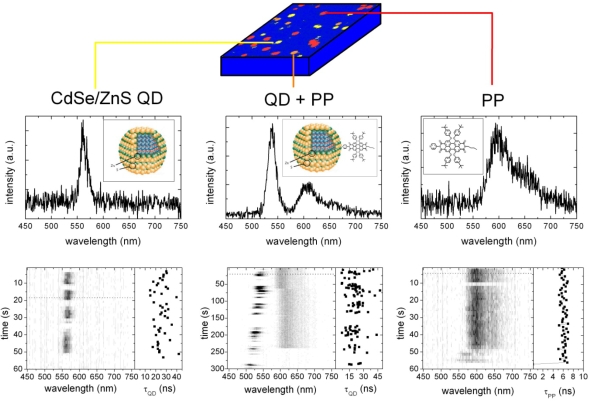
Luminescence spectra of single YQD and PP. Spectra are related to various confocal spots of the spin coated sample. Typical luminescence intensities, spectral positions and decay times are shown as a function of observation time of 60 s and longer.

Figure 4 shows spectra of single PP and YQD obtained via confocal microscopy for spin coated samples. The spectra can be divided into three classes, namely PP fluorescence (Figure 4 right), YQD photoluminescence (Figure 4 left) and the sum of the two spectra (Figure 4 middle). All types of spectra show typical single quantum object fingerprints, that is luminescence intermittency (blinking) [2,34] and spectral fluctuations. We show as a function of observation time on the right side below each spectrum the experimental luminescence decay time averaged over 500 ms. In case of PP we observe a nearly mono-exponential decay varying between 4 and 6 ns for different single molecules, while the decay time for YQD is even for a single QD fluctuating between 50 ns and 5 ns (our lower limit in time resolution).

The observation of the sum of the two spectra shown in the middle is been assigned to the formation of assemblies [26,32,35]. For as many as 50 single quantum objects we did not find a significant correlation between PP and YQD properties such as blinking or spectral shifts. It is also evident from the experiment that in case of bleaching of the dye (see Figure 4, middle) neither the YQD PL intensity is increasing, nor the PL decay time becomes longer, which would be the case if FRET would be the major quenching mechanism. Instead the quenching remains constant as is expected in case, when a NON-FRET process is the dominant quenching mechanism. Figure 4 also shows that during the time the dye molecule is in an “on”-state, the YQD PL intensity is subject to fluctuations. In blinking models [2,34] dark (or dim) periods correspond to strong radiationless decay processes, which are often much faster than FRET processes. This implies, that during “off” periods the PP fluorescence should slightly decrease due to the remaining but rather inefficient FRET process. To identify such effect we will correlate in future experiments YQD “on-” and “off-” PL periods with DTTP fluorescence intensities, as has been demonstrated recently for similar system with Cy5 dye molecules [35].

**Figure 5.**
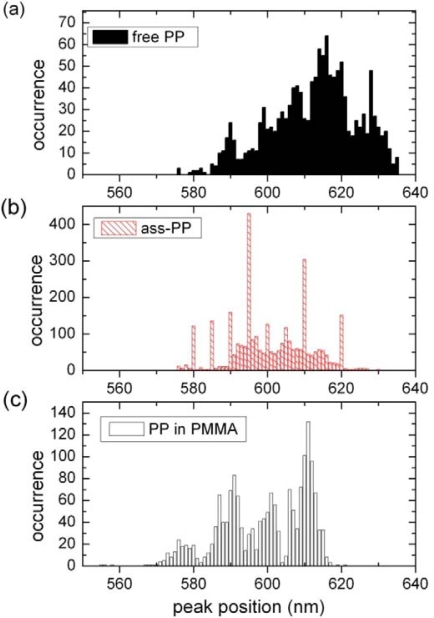
Distribution of fluorescence maxima of single molecules on SiO_2_ for 25 PP (a) and 25 PP in assemblies (b). For comparison distribution is shown for 30 PP in a spin coated PMMA film. (c). Fluorescence maxima have been obtained by fitting the PP fluorescence spectra by two Gaussian lines.

Individual PP molecules have different fluorescence spectra due to various conformations of the bay groups [6,40] resulting in a broad distribution of the fluorescence maxima. As we have shown recently [6], the corresponding distribution depends on the local environment such as the embedding matrix. Figure 5a shows a typical distribution of PP fluorescence maxima on a SiO_2_ surface, while the related distribution for YQD-PP assemblies is shown in Figure 5b. For comparison we show in Figure 5c the distribution for PP in a PMMA polymer film. Comparison of the distributions shown in Figure 5 reveals, that the assembly formation favors conformations in the short wavelength range becoming more equivalent to the distribution observed for PMMA.

**Figure 6.**
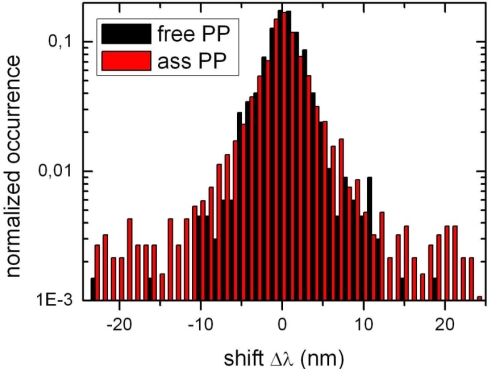
Spectral jumps for PP and YQD-PP during the respective complete observation time for a single PP molecule on SiO_2_.

As is evident from Figure 4 luminescence spectra of individual PP molecule are subject to spectral fluctuations. In Figure 6 we have plotted the individual spectral jumps of each PP during the complete observation time which is in the order of several seconds to several minutes both for PP and YQD-PP assemblies.

In the given example in Figure 4 the average YQD PL decay time is reduced from about 25 ns to 20 ns upon assembly formation. A similar decay time reduction has been observed for all of the assemblies and goes in line with the PL quenching upon assembly formation. Some YQD-AM show blue shifts, which are probably due to oxidation processes resulting in smaller crystals [36] at times longer than about 10 s. Blue shifts might also be due to a diffusion of shell atoms into the core of the QD [37]. Such a process might be accompanied by a shortening of the PL decay time and the PL intensity. We did not observe similar effects in case of TOPO ligands. Even more, ensemble experiments show that the blue shift, though present also in the absence of PBI, becomes enhanced in the presence of PBI and is most pronounced for DTPP.

**Figure 7.**
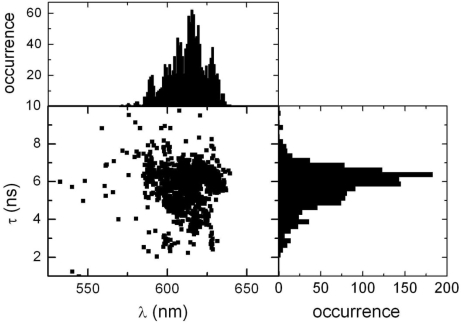
(bottom left) Correlation of PP fluorescence decay time and fluorescence emission wavelength λ (fluorescence maximum, t_bin_ = 1 s), (bottom right) distribution of fluorescence decay times and (top left) distribution of fluorescence maxima for immobilized PP on a glass substrate.

Though the PP fluorescence spectra and fluorescence decay times are rather stable for a given single molecule within the observation time, both vary among different PP. Figure 7 shows (lower left) the correlation between decay times and spectral positions. It can be seen, that the decay time is—if at all—decreasing with increasing wavelength. The plot on the right in Figure 7 shows the distribution of decay times, while the top left plot shows the wavelength maximum of the fluorescence spectra. For the decay times the observed range is between 2 and 9 ns, while the center is close to 6 ns.

## 3. Discussion

Figure 1 clearly shows that titration with PP dyes quenches the PL of YQD, while the PP fluorescence increases. As we have outlined earlier for other types of dyes [3,4] only part of the quenching is related to FRET. Comparing all ensemble experiments for the four different QD-dye systems the following observations emerge: (i) FRET is at most a 17% contribution to the total QD PL quenching. Due to the low FRET efficiency the total quenching has to be described by NON-FRET processes since CT can also be ruled out [5]. Accordingly, the observation of FRET on a single molecule level is almost not feasible and we will therefore concentrate on ensemble measurements. (ii) FRET efficiency is (as can be seen from Figure 2) larger for DTPP (TPP) as compared to DPP (PP) type dyes.

In the following we assume that assembly formation in toluene solution is a dynamic process influenced by the type and concentration of both ligands and dye molecules. Attachment of dye molecules needs the presence of accessible sites in the ligand shell and/or a replacement of a sufficiently large number of ligand molecules. Since the assembly formation is a complex thermodynamic process, the exact number of molecules on a QD surface is not known a priori.

In case that FRET has been analysed applying the Förster model while making use of the corresponding parameters for the nanoassemblies, it has been supposed [22–24,38] that the Förster theory is applicable by assuming the respective electronic dipole moments at the centre of the QD and the dye, respectively. In the following we like to discuss whether the specific aspects of the Förster model also apply to our present experiments.

**Figure 8.**
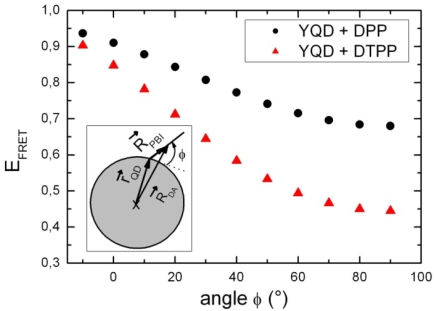
Calculated FRET efficiency for DPP and DTPP as a function of *φ*. The inset represents a scheme of our geometrical model. From geometrical constraints the orientation for DTPP corresponds to *φ* ≈ 0° and for DPP to *φ* ≈ 90°, respectively.

Comparing the two with respect to their geometry different types of coordinating pyridyl groups, DPP (and PP) can be attached for steric reasons to the QD surface by only one nitrogen lone pair (see Figures 8 and 10). In this case the long axis of DPP (PP) will be nearly perpendicular to the QD surface. For merely steric reasons, a perpendicular position of DTPP (TPP) will not be possible, instead a nearly tangential orientation of the long axis will be favoured (see Figs. 8 and 10). Such a qualitative argument can be tested by a simple calculation of the FRET efficiency (E_FRET_) using the standard Förster model [39]:

(3) $$E_{FRET}={\left(1+{\left(\frac{R_{DA}}{R_{0}}\right)}^{6}\right)}^{-1}$$

where Ro corresponds to the Förster radius and R_DA_ to the centre-to-centre donor-acceptor distance. While almost all relevant parameters are with respect to DPP and DTPP nearly the same, they differ considerably in ${\vec{R}}_{DA}={\vec{R}}_{PBI}+{\vec{r}}_{QD}$, the (vectorial) distance between the centers of the QD and the dye (R_PBI_ corresponds to the distance of the coordinating point to the centre of the chromophoric part of PBI while r_QD_ is the radius of CdSe/ZnS nanocrystal). For a hypothetical perpendicular position of both DPP and DTPP the distances become R_DA_(DPP) = 3.6 nm and R_DA_(DTPP) = 4.75 nm. This implies, that FRET should be stronger for DPP than for DTPP, which is quite opposite to what is observed experimentally (see Fig 2). However, taking into account the steric restrictions discussed above we calculated the FRET efficiency as a function of the orientation *φ* of the long molecular axis with respect to the QD surface as is shown in the inset of Figure 8. Now *E_FRET_* becomes 0.85 for DTPP at *φ* = 0° and 0.68 for DPP at *φ* = 90°, which is with respect to relative FRET efficiencies in rough agreement with the experimental result.

The parallel orientation of DTPP on the QD surface suggested by FRET results is schematically shown in Figure 10. A parallel orientation is reasonable, since the attachment of the terpyridyl unit to the QD surface needs the coordination of the two ortho-pyridyl groups (see Figure 10 (c), which can only be achieved in a flat and slightly drilled configuration of the terpyridyl unity. A direct consequence of this coordination to the surface metal ion is a spectral red shift observed for DTPP in case of the reduction of excess of TOPO from the solution (Figure 3). Whereas high excess of TOPO will favor a maximum surface coverage of YQD by ligands, a reduction of TOPO will diminish the coverage. This will allow for a more effective attachment of DTPP, which will result in a higher average number of DTPP on a single QD and thus a higher PL quenching. The accompanied spectral shift observed for DTPP might be explained by the fact that when passing from a high ligand coverage to a lower one DTPP–QD assemblies might be realized in such a way, that either the electronic π-system of DTPP couples directly with the electronic wave function of BQD, or DTPP molecules attached to the QD interact effectively (dimer formation). Another possibility is, that the distribution of conformations for DTPP is considerably changed. As has been reported recently [6,27] the conformations of DTPP are strongly related to the geometrical arrangement of the four bay groups.

Though relative FRET efficiencies can convincingly be related to various QD-dye assembly structures and to different relative orientation of the PBI dyes with respect to the surface, the surprisingly low FRET efficiency has to be discussed separately. To proceed, we have to discuss R_o_ which is defined as [39]:

(4) $$R_{o}=(\frac{9000\text{ln}10}{128\pi^{5}n^{4}})\kappa^{2}\varphi_{D}\;\cdot \;J_{DA}$$

where J_DA_ is the spectral overlap integral:

(5) $$J_{DA}=\int I_{D}(\lambda )\varepsilon_{A}(\lambda )\lambda^{4}d\lambda$$

**Figure 9.**
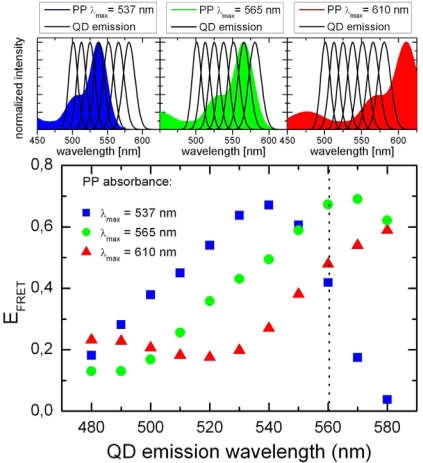
(top) Schematic spectral overlap for three PP conformations with the absorption maximum at 537 nm, 565 nm and 610 nm, respectively and QD PL between 500 and 580 nm. (bottom) FRET efficiency calculated for the respective conformations as a function of the QD emission wavelength.

*φ_D_* is the quantum efficiency of the donor and *κ*^2^ an orientation factor between donor and acceptor electronic transition dipole moments. Since QD have nearly spherical wave functions *κ*^2^ should vary between the dynamic (0.666) ore the static (0.476) random value [33]. The spectral overlap integral can be calculated from the corresponding absorption and emission spectra. The integral will for a given QD depend on the respective spectrum of the conformations of the PBI dye, which differ by spectral shifts of up to 60 nm [6]. The related spectral overlaps are shown in Figure 9, which clearly show that in the given range of the spectral distribution the FRET efficiency might vary between 0.4 and 0.7 for the YQD PL of 560 nm.

The initial quantum efficiency of YQD has been determined to be *φ_D_* = 0.6 and is reduced by a factor of 4 upon dilution. Taking *κ*^2^ = 0.476, *φ_D_* = 0.15 and a spectral overlap as given by the ensemble fluorescence spectrum of PP in toluene, we obtain a Förster radius of R_o_ = 4.1 nm. Following Equation (3) and applying the data above, R_0_ and hence the FRET efficiency may be reduced down to R_0_ = 2.7 nm and below E_FRET_ = 0.20, respectively.

While the results from ensemble experiments are most suitable to discuss overall FRET efficiencies they are less suited to discuss the specific structure of the assembly since PBI undergoes environment dependent conformation changes. However, single molecule data may provide more specific information on the structure of the assemblies. According to Figure 5 the spectral distribution of PP fluorescence is blue-shifted upon assembly formation. Such a shift indicates, that those conformations of PP are favored, for which the bay groups are most extended as this situation is related to short fluorescence wavelengths [6,27,40]. This corresponds to a situation where PP bay groups are as much within the molecular plane as possible. Such a situation is reasonable since PP has to be intercalated into the ligand shell which will be more easily accomplished for a nearly flat molecule, since less ligands have to be excluded from the QD surface. Thus the distribution is shifted from a “free” PP molecule on SiO_2_ [6] to a “matrix-isolated” type such as in PMMA (see Figure 5c)). At the same time Figure 6 reveals, that spectral fluctuations are enhanced for assembled PP as compared to PP on SiO_2_. From this we conclude, that the ligand shell imposes more flexibility than in PMMA [6] allowing for considerable conformational changes of PP. An alternative explanation is, that the curved QD surface allows for more conformational flexibility than the flat SiO_2_ substrate. The high flexibility of the PP phenoxy side groups on the QD surface is only enabled in absence of steric hindrance. Therefore (i) a nearly perpendicular geometry has to be established and (ii) the ligand density close to PP has to be low. In conclusion, the identification of PP conformations (i) has been used to confirm the surface geometry already suggested by FRET results and (ii) serves as probe for the ligand density of single quantum dots.

## 3. Experimental Section

**Figure 10.**
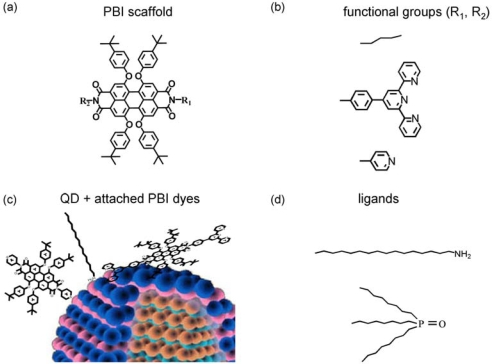
(a) Perylene bisimide (PBI) scaffold, (b) Functional units R_i_: alkyl (alk), terpyridyl (tpy) and pyridyl (pyr). For notations see Table 1. (c) Scheme of surface attachment of DPP and DTPP to CdSe/ZnS quantum dot (QD) (d) Amine and TOPO ligands.

CdSe QD are passivated by three monolayers of ZnS, resulting in a total diameter of 5.1 nm for the (yellow) QD (YQD, λ_abs_ = 542 nm) and 4.5 nm for the (blue) QD (BQD, λ_abs_ = 472 nm) with CdSe core diameters of 3.0 nm and 2.4 nm, respectively. For ensemble and single particle experiments core/shell quantum dots obtained from Evident Technologies are passivated by long-chain amines (AM) or trioctylphosphine oxide (TOPO) and were investigated in toluene solution of spectroscopic grade (Merck) with and without titration by differently functionalized perylene bisimide (PBI) molecules [41,42]. PBI molecules are shown in the Figures 10 a-b. For ensemble experiments an amount of 5 μL of a 60 μM solution of the CdSe/ZnS core/shell QD was diluted resulting in a 0.1 μM solution. Sample preparation and characterization follow the procedure as described recently [5].

To obtain molar ratios x = [PBI]/[QD] of about 0.1 to 10, volumes of 5–200 μL of the dye stock solutions were titrated. Due to the PL instability of QD in solution and in contrast to conventional titration experiments each increase of the molar ratio was carried out in a new cuvette with the same starting conditions to avoid intrinsic overlay of time dependent (quenching) processes. For some TOPO terminated BQD-dye samples the excess of TOPO in the solution has been reduced. Since the surfactants on the QD surface are in a dynamic equilibrium with those in solution [20] such TOPO depleted samples will result in a smaller surface coverage. After dilution of the QD the PL decays on time scales of several minutes as also reported in previous work [25]. After 40 minutes PL intensities remain almost constant also in case of AM surfactants. Now the dye solution is titrated to the QD solution by means of micropipettes. Single molecule/single particle experiments have been performed for QD-PP assemblies spin coated on a SiO_2_ substrate. Tentative assembly structures are shown in Figure 10 (c). The details of the experimental setup and procedures are described elsewhere [5].

## 4. Conclusions

Similar to previous experiments [3,4,31,32,43] we could show that QD-dye nanoassemblies can be formed via self-aggregation processes, in case that suitable functional pyridyl groups are able to coordinate the dye to the QD surface. In the present case we have used PBI dyes with two different pyridyl-type functional groups, that realize a geometrically well defined QD-dye assembly, which allows to determine the dependence of dynamic QD-dye interactions upon the spatial arrangement of the nanoassembly such as distance and orientation. Moreover, PBI dyes allow according to their high quantum yield and large photostability for single molecule detection. We therefore were able to compare dynamic interactions both by single molecule and ensemble experiments in solution on the base of a Förster-type energy transfer process. From a comparison of FRET for (YQD-DPP) and (YQD-DTPP) nanoassemblies we conclude, that DPP is oriented with the long axis nearly perpendicular to the YQD surface while DTPP prefers a more tangential orientation. However, it became also evident, that the FRET efficiency is quite low which we assign to a considerable reduction of quantum yield of QD upon the extreme dilution. Moreover, NON-FRET processes are stronger than FRET. The observation has to be taken into account when investigating PL quenching upon assembly formation. Comparing experiments on isolated single PP molecules with those on single aggregates allows for interpretations of changes of the PP conformations upon assembly formation, that is increase of PP “flatness” and increase of interchange between conformations.

## Figures and Tables

**Table 1.** Notation for PBI molecules.

**Abbreviation**	**R_1_**	**R_2_**

PP	pyr	alk
DPP	pyr	pyr
TPP	tpy	alk
DTPP	tpy	tpy
